# Role of hypoxia-inducible factor – 1 alpha on the progression of cervical intraepithelial neoplasia and cervical cancer: a narrative review

**DOI:** 10.3389/fonc.2026.1922814

**Published:** 2026-09-10

**Authors:** Yenddy N. Carrero, Jesús A. Mosquera

**Affiliations:** 1 Faculty of Health Sciences and Human Development, Universidad Ecotec, Samborondón, Ecuador; 2 Instituto de Investigaciones Clínicas “Dr. Américo Negrette”, Facultad de Medicina, Universidad del Zulia, Maracaibo, Venezuela

**Keywords:** angiogenesis, biomarker, cervical cancer, hypoxia, lesion, metastasis, progression, tumor microenvironment

## Abstract

Cervical cancer represents a global public health problem, hence the importance of optimizing therapeutic options and expanding knowledge of prognostic markers. Clinical and experimental evidence shows that hypoxia-inducible factor (HIF) is a key inducer of aggressive phenotypes in cervical cancer: promoting angiogenesis, invasion, epithelial-mesenchymal transition (EMT), modulation of the immune microenvironment, and contributing to resistance to radiotherapy/chemotherapy in a possible synergistic effect with the human papilloma virus oncogenes. Hypoxic microenvironments that modulate the early activation of HIF pathways may be present in pre-invasive lesions and favor the acquisition of invasive phenotypes that precede invasion. This review highlights the mechanisms of HIF-1α in the progression of cervical intraepithelial neoplasia and cervical cancer. In this regard, HIF-1α is involved in the expression of genes related to angiogenesis, apoptosis and progression such as GLUT1, uPAR, BIRC5, EPO, EpoR, LIMD1, VHL, MET and pro-angiogenic genes (VEGF, PGF, PDGF-β, PAI-1, MMP-2, MMP-9, ANG-1, ANG-2, using pathways such as YAP/TA2. However, the balance between antitumor factors (miR-143) and protumor factors can define the progression of this neoplasia. In the future, the characterization of hypoxia signatures and the evaluation of HIF-1α pathway inhibitors are promising tools for optimizing outcomes in the treatment of cervical cancer, integrated with modern, high-precision radiation therapy techniques and predictive models based on imaging and artificial intelligence, which represent a key pillar of personalized oncology in cervical cancer, with the potential to improve tumor control and reduce toxicity; however, specific clinical trials are still needed to validate their impact on survival and safety.

## Graphical Abstract

**Figure:**
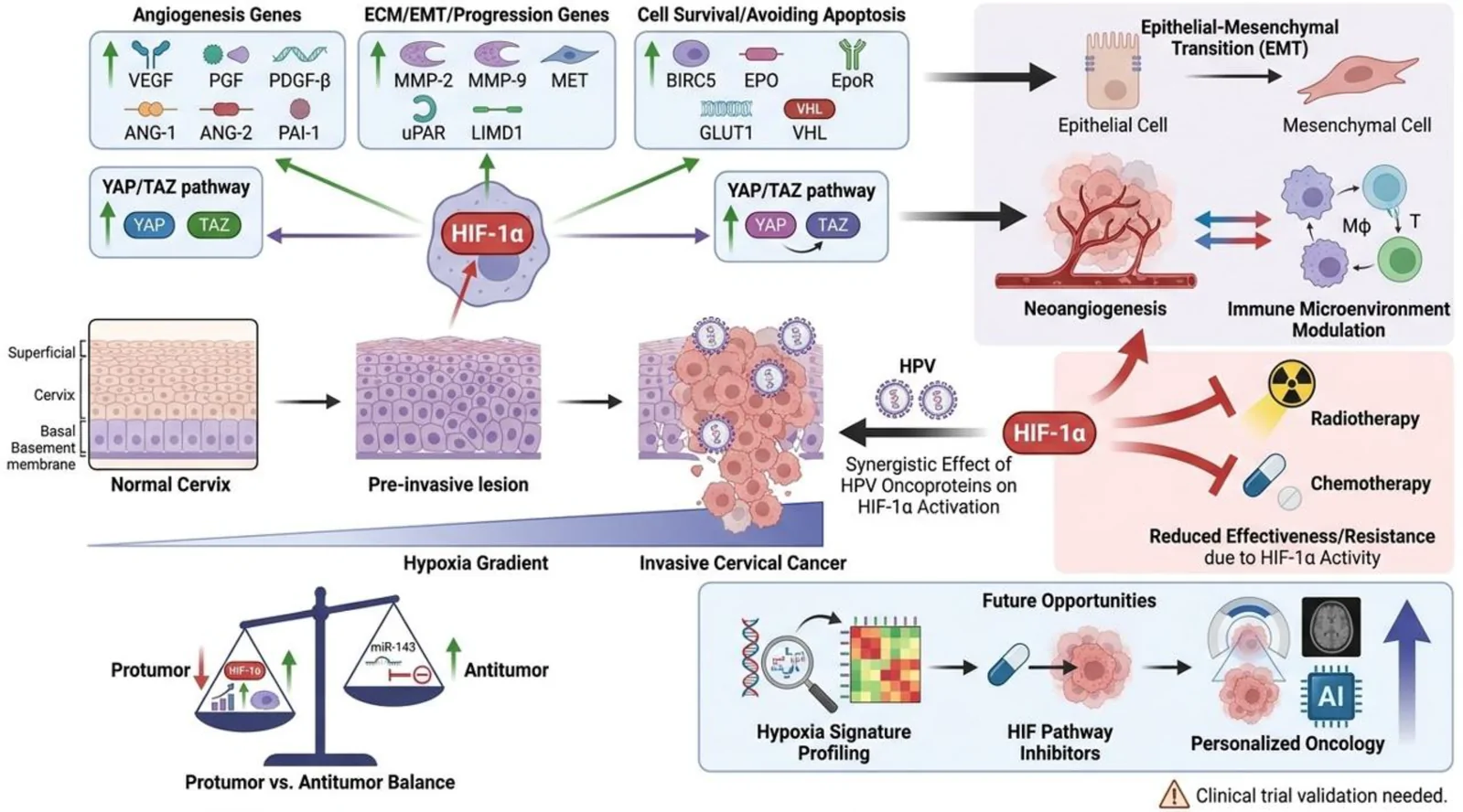
Hypoxia–HIF-1α axis in cervical cancer progression and therapeutic resistance. Hypoxic microenvironments in pre-invasive cervical lesions and invasive tumors stabilize HIF-1α, promoting transcriptional programs associated with tumor progression. HIF-1α contributes to angiogenesis, invasion, epithelial–mesenchymal transition (EMT), immune microenvironment modulation, and resistance to radiotherapy and chemotherapy, potentially in synergy with HPV oncogenes. This pathway is associated with altered expression of genes involved in metabolism, survival, invasion, and angiogenesis, including GLUT1, uPAR, BIRC5, EPO, EpoR, LIMD1, VHL, MET, VEGF, PGF, PDGF-β, PAI-1, MMP-2, MMP-9, ANG-1, and ANG-2, as well as related signaling pathways such as YAP/TAZ. The balance between protumor hypoxic signaling and protective regulators such as miR-143 may influence progression from cervical intraepithelial neoplasia to invasive cervical cancer. Hypoxia signatures, HIF pathway inhibitors, advanced radiotherapy, and imaging- or artificial intelligence-based predictive models are promising strategies for personalized treatment, although dedicated clinical trials are still needed to confirm survival benefit and safety. Created with the Figure Labs (2026) artificial intelligence tool.

## Introduction

Cervical cancer represents a global public health problem, being the fourth most common cancer and the fourth leading cause of cancer death in women. In 2022, ~662.300 new cases of cervical cancer and ~348.900 deaths were reported globally. The age group of women ≥ 65 years in 2022 represented 157.200 cases and 124.300 deaths, while for women under 45 years, cervical cancer is among the 3 most common cancers in 149 countries and one of the 3 leading causes of death in 154 countries, hence the importance of expanding the study of all the mechanisms of progression, treatment efficacy and therapeutic targets (1).

Various risk factors in conjunction with persistent infection with high-risk types of human papillomavirus (HPV), particularly HPV-16 and HPV-18, are necessary but not sufficient etiological factors. Other factors of the tumor microenvironment (TME) such as hypoxia play a key role in the progression and aggressiveness of cervical cancer. TME in addition to genetic and environmental factors plays an important role in the progression of malignant tumors, promoting tumor genesis, progression, metastasis and deterioration through different mechanisms (2). Hypoxia is a feature of the TME that correlates with adverse clinical findings in various solid tumors (3–5). Hypoxia affects the function of cytotoxic T cells and generates the recruitment of regulatory T cells, decreasing tumor immunogenicity (6). Additionally, hypoxia can stimulate a complex cellular signaling network in transformed cells, including the hypoxia-inducible factors (HIF), phosphoinositide 3-kinase (PI3K), mitogen-activated protein kinase (MAPK) and nuclear factor kB (NF-kB) pathways, which are able to interact with each other and generate positive and negative feedback loops that increase or decrease hypoxic effects (7, 8).

Pre-invasive lesions of the cervix, known as cervical intraepithelial neoplasia (CIN-1, CIN-2, CIN-3), represent a group of histopathological alterations that encompass a spectrum of progressive changes in the epithelium, progressing to carcinoma *in situ* prior to invasive cancer. The proportion of cervical epithelium with dysplastic cells determines the degree of dysplasia. CIN-1 (low-grade) affects the lower third or less of the epithelium, while CIN-2 and CIN-3 (high-grade) progress to involve the entire thickness of the epithelium (9). It is important to note that regression can occur in some types of lesions; therefore, progression rates to cancer are variable. While persistent HPV infection plays a significant role in malignancy, the immune system eliminates most HPV infections. The risk of CIN increases with the HPV type involved, especially types 16 and 18, which are high-risk viruses (10). In addition to HPV infection, the activation of cellular pathways that promote angiogenesis, resistance to apoptosis, and adaptation to hypoxia are also involved. In this context, hypoxia-inducible factor-1 alpha (HIF-1α) acts as a regulator that promotes the expression of proangiogenic genes such as VEGF, facilitating the transition from lesions to invasive carcinoma (11). In this regard, at reduced oxygen tensions, HIF 1 and 2 are stabilized and mediate a hypoxic response, primarily as transcription factors, exerting differential effects on tumor growth and affect important features of cancer, including cell proliferation, apoptosis, differentiation, vascularization/angiogenesis, and cell death (12).These factors are classified as factors-1 alpha (HIF-1α) and HIF-2α (13) which possess contrasting functions, as demonstrated by the opposite regulation of c-Myc (14). HIF-1 α overexpression has been associated with poor prognosis, metabolic reprogramming, immune evasion and resistance to treatment (11, 15). HIF-1 α is a heterodimer composed of 2 subunits, HIF-1α and HIF-1β. As a transcription factor, HIF-1α can transactivate more than 70 target genes, which are oxygen-dependent and capable of regulating hypoxic gene expression and signal transduction pathways. HIF-1α is a key regulator of erythropoiesis, angiogenesis, cellular metabolism and genomic stability (16) and involved in cancer (17). In various types of cancer, such as breast, head and neck, cervical, and sarcoma, regions of tumor hypoxia are found, resulting in increased expression of HIF-1α and HIF-2α proteins in response to the hypoxic environment. These transcription factors drive a complex network of genes that drive multiple phases of cancer progression, such as angiogenesis and metabolic reprogramming through extracellular matrix (ECM) remodeling, cancer stem cell (CSC) specification, epithelial-mesenchymal transition (EMT), and immune response evasion (18). These factors prevent cell death, stimulating cell proliferation or inducting apoptosis, by mechanisms depend on oxygen concentration and ATP level. Hypoxia can inhibit the electron transport chain in mitochondria, triggering activation of Bax or Bak, and leading to cytochrome C release and activation of caspase-9 towards apoptosis (7).

Previous clinical studies have shown that patients with hypoxic cervical cancers had a significantly worse prognosis compared with patients who had better oxygenated tumors regardless of the therapeutic regimen they received (19, 20). A critical aspect that stands out in cervical cancer is the heterogeneity in clinical outcomes in patients with the same disease (21), a fact that highlights the need to establish markers that can predict patient outcomes and molecular tools that allow personalized treatment. A wide range of treatments has been employed for cervical intraepithelial neoplasia (CIN) and cervical cancer. With the aim of avoiding the collateral damage associated with chemical drug therapies, research has been conducted on the anti-cancer effects of plant-derived products in cervical neoplasia cell lines such as ME-180, SiHa, and HeLa (22, 23). This study aims to understand the molecular chronology of cervical carcinogenesis by highlighting the progressive overexpression of HIF-1α in a hypoxic microenvironment and early tumor adaptation. Therefore, relevant information will be included that considers all spectra of cervical lesions, which could be used in future studies for its predictive and prognostic value, as well as for the design of targeted therapies.

## Methods

### Databases consulted

A narrative review was conducted; to identify the evidence, a systematic search was performed in the PubMed/MEDLINE, Embase, Scopus, and LILACS databases, using a combination of controlled vocabulary descriptors (MeSH and Emtree terms) and free-text terms, linked by Boolean operators.

### Search strategy and Boolean operators

Terms in Spanish and English were used in combination with the Boolean operators AND, OR, and NOT. The strategy combined three conceptual blocks: the first related to the hypoxia-inducible factor (“Hypoxia-Inducible Factor 1, alpha Subunit” [Mesh], HIF-1, HIF1A, HIF-2, HIF2A, EPAS1); the second related to cervical neoplasia and its precursor lesions (“Uterine Cervical Neoplasia” [Mesh], “Uterine Cervical Dysplasia”[Mesh], cervical intraepithelial neoplasia, CIN, SIL), and the third related to tumor progression and its outcomes (“Disease Progression”[Mesh], “Neoplasia Invasiveness”[Mesh], invasion, metastasis, prognosis, biomarker*), linked together using the AND operator. The search was restricted to studies in humans, with no language restrictions beyond English, Spanish, and Portuguese, and was supplemented by a manual review of the reference lists of the included articles and previously identified systematic reviews, to identify additional studies not captured by the electronic search strategy.

### Search period

The literature search was limited to the period from January 2016 to July 2026, a time frame justified by two methodological considerations. First, it allows us to capture the consolidation of evidence regarding the mechanistic role of HIF-1α as a mediator of the metabolic reprogramming induced by the HPV oncoproteins E6 and E7; and second, it incorporates the period during which the convergence between HIF signaling and immune checkpoints, as well as certain therapeutic options, has been documented. No additional date restrictions were imposed on seminal studies on the molecular characterization of HIF-1α prior to 2016 when these were consistently referenced in the most recent literature included in the review, given their value as a conceptual foundation for the development of this field.

### Inclusion criteria

The following were included:

1. Human studies (tissue, human cervical cell lines, or clinical cohorts) that evaluate the expression, activity, or modulation of HIF-1α and/or HIF-2α in the cervix.
2. Studies that include at least one comparison between stages of disease progression (normal tissue vs. CIN, low-grade vs. high-grade CIN, or preinvasive lesion vs. invasive carcinoma) or correlation with clinical outcomes (survival, response to treatment, recurrence).
3. Eligible study designs: cohort studies, case-control studies, cross-sectional studies with immunohistochemical or molecular analysis, *in vitro*/*in vivo* experimental studies using a cervical model, and previous systematic reviews and meta-analyses (for triangulation of references, not for primary data extraction).
4. Publications in English, Spanish, or Portuguese.
5. Publications with full text available.
6. Articles published in indexed, peer-reviewed journals.

### Exclusion criteria

1. Studies focusing exclusively on HIF in other tumor types without a cervical component
2. Letters to the editor, conference abstracts without extractable data, editorial comments, and opinion pieces without original data
3. Studies with diagnostic ambiguity
4. Studies with an unreported sample size or unspecified HIF measurement methodology
5. Preprints that have not undergone peer review.

### Hypoxic environment and hypoxia-inducible factors

Several studies have described hypoxic micro-regions in a wide variety of solid tumors, originating in different tissues, such as uterine cervix, head and neck, breast (24), pancreas (25), prostate (26), larynx (27), endometrium (28), lung (29), as well as soft tissue sarcomas (30). The mechanisms by which sustain tumor hypoxia can increase cancer aggressiveness with adverse outcomes include differential regulation of gene expression and clonal selection of tumor cells that have lost their apoptotic potential (31). Due to the rapid proliferation of a growing tumor mass coupled with the aberrant architecture of microvessels, the cells that make up solid tumors are prone to oxygen deficiency. In cancer biology, hypoxia confers a greater propensity for chemoresistance, radioresistance, disease relapse, and enhances the metastatic process (32). Therefore, the accelerated proliferation of tumor cells and the irregular development of tumor vasculature are the direct causes of the onset of hypoxia (33). Hypoxia also promotes M2 polarization of macrophages, favoring the pro-tumorigenic effects of myeloid-derived suppressor cells, and induces the differentiation of CD4-positive T lymphocytes into immunosuppressive regulatory T lymphocytes (34). Thus, oncological hypoxia resulting from a combination of acute, chronic and systemic forms of hypoxia, can promote tumor progression and is considered one of the main causes of resistance to various therapeutic modalities (35, 36), chronic hypoxia being the most common form of hypoxia in tumor tissues (37).

One characteristic of tumors is their ability to adapt and proliferate in conditions of hypoxia, which is lethal to normal cells. In this sense, HIFs have dual action, being vital for normal cell function but potentially dangerous when absorbed by cancer cells. When HIF is activated in cancer cells, it induces the transcription of genes that modify the metabolic profile from oxidative phosphorylation to a more glycolytic phenotype, and promotes other survival mechanisms, notably angiogenesis (2). In this regard, the upregulation of HIF-1α, which can activate target genes related to erythropoiesis, glycolysis, and angiogenesis play an important role. In addition, HIF-1α can regulate the expression of genes that encode growth factors and receptors, the apoptotic pathway, cell cycle regulators, invasiveness, and metastasis (38, 39). In this regard, HIF-1 targets genes associated with numerous growth factors, notably transforming growth factor α (TGFα) and insulin-like growth factor 2 (IGF2) (40). The interaction of these molecules with their specific receptors, such as the epidermal growth factor receptor (EGFR) and the insulin-like growth factor 1 receptor, activates signal transduction pathways that result in the synthesis of HIF-1α and promote cell survival and proliferation (39).

In healthy cervical tissue, the average oxygen concentration varies from ~5% to 6% O_2_ (physoxia) but is reduced to ~1.2% O_2_ in cervical cancers (41). Chronic hypoxia has generally been described as an important aspect of carcinogenesis, caused by limitations in oxygen diffusion, restricting the supply of O_2_ to cancer cells that are not near the tumor’s blood vessels. However, there is another type of hypoxia, cyclic hypoxia (cycH), which appears due to repeated limitations of perfusion in tumor microvessels, which are often structurally impaired, inducing variations in erythrocyte flow. Recurrent phases of hypoxic (H) and reoxygenation (R) states (H-R cycles) occur in nearby tumor cells, with variable frequency and duration (42–44). There is evidence that cycH could select subpopulations of cancer cells that are even more aggressive and resistant to treatment than chronic hypoxia (43, 45).

Hypoxia shapes the local immunosuppressive TME, limiting the efficacy of antitumor immune responses (46). Hypoxia may induce the secretion of proteins by tumor cells, primarily secreted in exosomes, with the potential to remodel the TME, justifying the importance of finding strategies that can efficiently reprogram the immunosuppressive hypoxic TME. Just as the hypoxia-inducing factors, HIF-1α and HIF-2α can regulate the expression of immune checkpoints, tumor cells evade the innate immune response through a cell surface protein that interacts with signal-regulatory protein α (SIRPα) on the surface of macrophages to block phagocytosis (47, 48). Recent studies found that hypoxia increased zinc finger E-box binding homeobox 1(ZEB1) expression in cervical squamous cell carcinoma (CSCC) cells, resulting in increased tumor-associated macrophage (TAM) infiltration, and that hypoxia-induced ZEB1 expression is correlated with CD47-SIRPα axis activity in CSCC, conferring to cancer cells the ability to evade macrophage phagocytosis promoting tumor progression (49).

Hypoxia as described affects the mechanisms of apoptosis, and when cells that have lost their apoptotic potential are propagated by the decrease in p53 expression, the increase in the expression of the mutant protein p53 or Bcl-2, the increase in angiogenesis and the general increases in proteinase activity, there is a greater risk of metastasis (50). Experimental studies in lung cancer have shown that the main function of wild type p53 gene, (wt p53) is to modulate the cell cycle and accelerate cell death, and that the physical binding of HIF-1α to wt p53 facilitates wt p53-mediated hypoxic cell death. Therefore, hypoxia-mediated activation of HIF-1 leads to the formation of a mutant p53/HIF-1 complex that physically binds to the SWI/SNF chromatin remodeling complex, promoting the expression of a selective subset of hypoxia-sensitive genes. Depletion of p53 mutants alters HIF-mediated upregulation of extracellular matrix (ECM) components, affecting the tumorigenic potential of NSCLC (non-small cell lung cancer) cells *in vitro* and *in vivo* in murine models (51).

**Figure 1:**
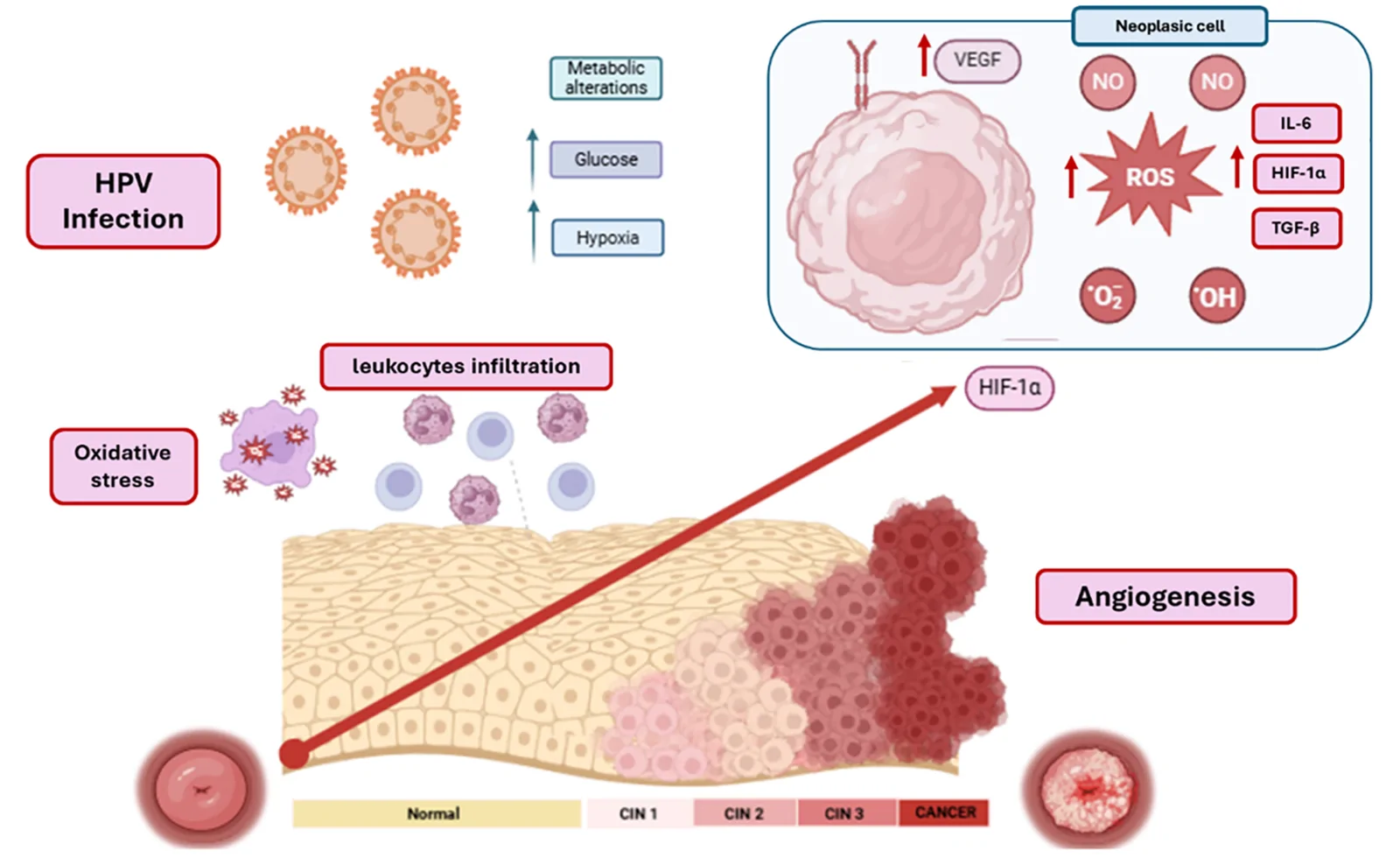
Under hypoxic conditions, HIF1A activation induces a series of signaling events, including the activation of the programmed death receptor-1/programmed death ligand-1. In addition, it triggers the release of class I chain-associated complex molecules by altering nitric oxide signaling, which alters immune surveillance by natural killer cells. An increase in HIF1A expression directly proportional to the degree of cervical lesion has been evidenced; moreover, the E6 and E7 oncoproteins of high-risk HPV not only act by inactivating p53 and Rb but can also modulate the HIF-1α pathway. Therefore, HIF-1α not only participates in the reduction of the efficacy of radiotherapy, chemotherapy and targeted therapies, but its target genes are also involved in a multitude of pathophysiological mechanisms, such as the regulation of angiogenesis, cell proliferation and survival, glucose metabolism, immune escape and iron metabolism.

Understanding tumor hypoxia is critical for new prognostic and treatment insights. Hypoxia-related genetic signatures have been developed that predict overall survival in cervical cancer. A recent study identified and validated a five-gene prognostic signature, with experimental verification of its potential as potential prognostic targets (52). Hypoxia detection in tumors is challenging because levels of HIF and hypoxic target proteins can be altered during tissue fixation; furthermore, native samples with preserved O_2_ supply are not readily available. Therefore, detection of HIFα in combination with CAIX (carbonic anhydrase IX), GLUT1(Glucose Transporter 1), or VEGF (vascular endothelial growth factor) is recommended (53). It was shown that high expressions of HIF1α and low expressions of HIF2α or a “HIF index” are poor prognostic factors, when overexpression of CAIX, GLUT1 and GAPDH (GAPDH glyceraldehyde-3-phosphate dehydrogenase) follow this prognostic trend (54) (**Figure 1**).

### Hypoxia-inducible factors and oxygen reactive species

Tumor cells produce various cytokines and chemokines that attract a diverse population of leukocytes consisting of neutrophils, dendritic cells, macrophages, eosinophils, lymphocytes, and other cells, which are capable of producing a variety of cytokines, cytotoxic mediators including reactive oxygen species, serine and cysteine ​​proteases, MMPs, membrane-perforating agents, soluble killer cell mediators, tumor necrosis factor-alpha, interleukins, and interferons. The number and type of leukocytes present in infiltration is related to the cytokines produced by tumor and stromal cells located at the tumor site (55, 56).

Prolonged oxidative stress leads to the formation of free radicals that contribute to a chronic inflammatory environment that facilitates the process of carcinogenesis. Oxidative stress can lead to the activation of several transcription factors, such as NF-κB (Nuclear factor kappa B), AP-1 (Activator protein 1), p53, HIF-1α, PPAR-γ (Peroxisome proliferator-activated receptor gamma), β-catenin/Wnt and Nrf2 (Nuclear factor erythroid 2-related factor 2) facilitating the release of a variety of inflammatory mediators (55, 57).

Previous studies have reported that nitric oxide (NO•), hypoxia-inducible factor-1 (HIF-1) and decreased actin expression play a role in cancer progression (58). It has also been described those alterations in action, NO•, a vasodilator, has been shown to play a role in the regulation of cancer progression and metastasis; such effects are probably mediated by angiogenesis, apoptosis and cell motility (59, 60). In addition, NO• increases HIF-1, a transcription factor that potentiates several hypoxia-inducible genes and induces the metastatic process (61). Under hypoxic conditions, HIF activation causes lactic acid accumulation, a metabolic feature of various tumors. Cancer cells, regardless of changes in oxygen concentration, also exhibit the Warburg effect aerobic glycolysis or the aerobic glycolytic phenotype (62). These metabolic rearrangements at the expense of HIF can induce microenvironmental alterations, such as lactate production and acidification, which are important oncogenic characteristics (63). Angiogenesis is the growth of new blood vessels and is essential for tumor progression and metastasis (64). It has been described that increased activity of the isoform of the enzyme iNOS (inducible nitric oxide synthase) facilitates angiogenesis and metastasis of tumor cells, probably mediated by interleukin-33 (65).

There are three pro-survival pathways that synergistically promote the transcription and translation of HIF-1α, especially in cancer: Phosphoinositide 3-OH kinase/protein kinase B/mechanistic target of rapamycin (PI3K/Akt/mTOR), extracellular signal-regulated kinase/mitogen-activated protein kinase (ERK/MAPK), and Janus kinase/signal transducer and activator of transcription (JAK/STAT) (66). HIF-1α expression has been shown through both transcriptional and translational upregulation when it activates the PI3K/Akt/mTOR pathway. This activation occurs due to the accumulation of reactive oxygen species (ROS) and reactive nitrogen species induced by mitochondrial DNA (mtDNA) mutations, intermittent hypoxia, inflammatory mediators, and chemical toxins (67).

This is a relevant aspect in the progression of cervical cancer. The dynamic expression of circular RNAs (circRNAs) in cervical cancer progression was characterized, identifying circPOLD1 as a new stage-specific circular RNA that shows a progressive increase from normal cervix to high-grade squamous intraepithelial lesions, making it a promising candidate for early detection and diagnosis (68).

This being a relevant aspect in the progression of cervical cancer. A study showed that a ROS-generating mtDNA mutation in the MT-ND6 (NADH-ubiquinone oxidoreductase chain 6 protein) gene induces overexpression of HIF-1α mRNA, leading to increased production of HIF-1α protein and VEGF under hypoxic conditions, and suggesting the possibility that some pathogenic mtDNA mutations may activate HIF-1α transcription (67). Incubation of cells with H2O2 or an oxidative stress factor leads to stabilization of HIF-1α protein and activation of HIF target genes under normoxic conditions (69, 70). There is evidence that 1.5% hypoxia initially causes a burst of mitochondrial ROS leading to stabilization of HIF-1α (71, 72). At 1.5% oxygen, mitochondria act as O_2_ sensors stabilizing HIF-1α and releasing ROS from complex III into the cytosol. Furthermore, HIF-1α can be stabilized by oxidant-induced covalent modifications due to S-glutathione at its Cys520 residue, which inhibits ubiquitination by the von Hippel-Lindau (VHL) tumor suppressor protein (73). These series of oxidative changes lead to the accumulation of HIF-1α and its translocation to the nucleus, where it forms the HIF-1αβ complex, which is recruited to the hypoxia response element (HRE). This leads to the upregulation of several genes involved in immune response, energy metabolism, iron metabolism, glucose metabolism and transport, cell proliferation and survival, and angiogenesis (74, 75). Moderating ROS levels are of great importance for cell survival, proliferation, differentiation and migration, facts that give ROS a crucial role in the context of cancer (76, 77). In this regard, high oxidative stress can be reduced by increasing antioxidant molecules, such as the glutathione and thioredoxin systems that can reduce the level of H_2_O_2_ using NADPH, which is relevant in biosynthetic processes and in the regulation of ROS levels (78).

NO• has an important role in various stages of the carcinogenesis process, including DNA damage, activation of oncogenes, inhibition of DNA repair enzymes and tumor suppressor genes, as well as modulation of apoptosis and metastasis (79, 80). As described above, there is a relationship between HIF expression and angiogenesis, highlighting the role played by HIF-1α in the induction of iNOS, which leads to an increase in NO•, which has been shown to increase angiogenesis and progression to metastasis in various types of tumors (81, 82). S-nitrosylation is the covalent and reversible modification of a cysteine ​​thiol group in a protein by NO•. There are redox-sensitive transcription factors such as hypoxia-inducible factor estrogen receptor-1 and NF-κB that are regulated by S-nitrosylation. Signaling events regulated by S-nitrosylation can lead to cancer progression or inhibition, as S-nitrosylation of NF-κB and matrix metalloproteinase 9 (MMP9) stimulate cell death, while S-nitrosylation of caspase-3, caspase-9 and the N-terminal c-Jun kinase prevents activity by inhibiting apoptosis (79, 83) (**Figure 1**).

### Hypoxia-inducible factors and angiogenesis

Tumors are characterized by developing the ability to adapt and proliferate under conditions of hypoxia, which is lethal to normal cells. HIF is critical in enabling adaptability. In cancer cells, HIF induces the transcription of hundreds to thousands of genes that modify the metabolic profile from oxidative phosphorylation to a more glycolytic phenotype, and promote other survival mechanisms, such as angiogenesis, thereby increasing the tumor’s blood supply (2, 84). Hypoxia-inducible factor can induce the expression of various proangiogenic factors, including VEGF with its corresponding receptors platelet-derived growth factor B (PDGF-B), plasminogen activator inhibitor-1 (PAI-1), TIE-2 receptor, matrix metalloproteinases (MMP-2 and MMP-9) and angiopoietins (ANG-1 and ANG-2). VEGF-A is considered a potent endothelial mitogen, and one of the most relevant in the carcinogenic process, being expressed in various tumors (7, 85). There is evidence that VEGFA is an important downstream factor of HIF-1 (86), demonstrating that it plays a key role in tumor angiogenesis and vascular mimicry (87). The study by Peng et al. (88) found that following the overexpression of JAM3, a member of the immunoglobulin (Ig)-like JAM family, currently considered a tumor suppressor in some types of cancer, the expression of HIF-1α and VEGFA mRNA and protein is significantly overexpressed. They concluded that JAM3 overexpression activates the HIF-1α/VEGFA pathway, demonstrating that JAM3 promotes the migration and invasion of cervical cancer cells by activating the HIF-1α/VEGFA pathway.

A relevant aspect in the development of cervical cancer is angiogenesis, with certain unique differences compared to other solid tumors. Normal stratified epithelia, including the cervix, have characteristics of chronically hypoxic tissue, such as HIF-1α stabilization and VEGF expression, which increase further during progression to malignancy, with evidence of increased vascularity even in low-grade lesions, an event that occurs later in many other types of cancer (55, 58, 89–92). Greater vascularity is observed in low- risk HPV infections of the cervix, whereas this is usually a late event in the progression of many other cancers. In cervical carcinoma, intratumoral hypoxia leads to the stabilization of HIF-1α, which allows the transcription of proangiogenic genes, one of the most relevant beings. A study by Wang et al. (93),, observed that the expression of HIF-1α, GLUT-1, and VEGF in uterine cancer is significantly higher than in normal cervical epithelium. They concluded that combined detection could serve as a reliable indicator for assessing cervical cancer malignancy and predicting the efficacy of clinical radiotherapy.

An interconnection of hypoxia and α-ketoglutarate (α-KG) through HIF hydroxylases has been described. These enzymes act as oxygen sensors and depend on α-KG to carry out their function. Hypoxia affects oxygen availability, which in turn influences the activity of these hydroxylases. Low levels of α-KG can inhibit the degradation of HIF-1α and enhance the process of angiogenesis and tumorigenesis. Inferring that decreased α-KG related to IDH mutation stabilizes HIF-1α and leads to aberrant cell proliferation (84, 94).

Various research projects seek to target the HIF-1 pathway and its interrelated mediators, which would prove to be an excellent strategy for cancer prevention and treatment. Therefore, efforts have focused on developing multitargeted therapies that modulate dysregulated pathways in cancer angiogenesis, invasion, and metastasis (95) (**Figure 1**).

### Hypoxia-inducible factors and papillomavirus infection

Human papillomavirus (HPV) is a member of the papovaviridae family and is a relatively small, non-enveloped virus, about 55 nm in diameter. It has an icosahedral capsid with 72 capsomers containing at least two capsid proteins, L1 and L2. Each capsomere is a pentamer of the main capsid protein, L1 (96). The HPV genome consists of a single circular double-stranded DNA molecule with all the protein-coding sequences of the Open Reading Frame (ORF) confined to one strand, in addition to three functional regions in the genome (97).

The causal relationship between HPV infection and cervical cancer is well known. HPV infection is the leading cause of cervical cancer, and it is estimated that 99% of cases of cervical cancer are associated with persistent HPV infection, especially the high-grade oncogenic genotypes (98). The virus infects the mucocutaneous epithelium and produces viral particles in mature epithelial cells, causing disruption of normal cell cycle control and promotion of uncontrolled cell division leading to accumulation of genetic damage (98). A complex bidirectional relationship has been established between HPV and hypoxia, which is mediated by HIF. HPV can affect cellular signaling pathways to promote tumor growth, while hypoxia in turn affects the expression of viral oncoproteins in the tumor microenvironment, generating significant effects on cancer progression and response to treatment (99).

HPV infections have been categorized as infections by “low-risk HPV,” “high-risk HPV,” and “mixed HPV.” Specifically, the “mixed HPV” category includes any combination of high and low-risk genotypes (100). This grouping may inadvertently obscure the true incidence and clinical implications of high-risk HPV infections in the studied population. According to the National Cancer Institute (NIH), high-risk carcinogenic HPV genotypes include 12 types: HPV 16, 18, 31, 33, 35, 39, 45, 51, 52, 56, 58, and 59 (101). Among these, HPV 16 and HPV 18 are recognized as the most frequent carcinogenic HPV-related genotypes related to CIN/cancer. The presence of a single high risk HPV genotype already confers a significant health risk, regardless of coinfection with low-risk types. Thus, the classification into high and low risk is related to the frequency of the association between HPV and the pathology, in this case, CIN/cancer. Studies have shown that the high-risk HPV oncoproteins E6 and E7 not only act by inactivating p53 and Rb but can also modulate the HIF-1α pathway. Thus, E6 can stabilize HIF-1α indirectly by inhibiting its p53-mediated proteasomal degradation, while E7 can increase transcription of HIF-1 target genes (99). The oncoprotein E7 is associated with increased HIF-1α expression via ROS, ERK1/2, and NF-κB (102). Meanwhile, oncoprotein E6 positively regulates HIF-1α, preventing its ubiquitination by VHL and subsequent degradation by the proteasome (103). Therefore, both oncoproteins could indirectly promote increased glycolysis and resistance to intracellular pH variations through increased expression of HIF-1α and its target genes, such as GLUT1, LDHA, CAIX, MCT4, and BSG (104, 105). This synergy arising between viral infection and hypoxia contributes to an increased tumorigenic capacity.

HIF-1α has been found to be overexpressed in cervical cancer precursor lesions, associated with progression to cervical cancer (16). HIF-1α plays a significant role in infectious and inflammatory diseases. The immune response in carcinogenic processes such as those in the cervix, where viral oncoproteins are generally expressed, produces various cytokines and chemokines that attract a diverse population of infiltrating leukocytes, such as neutrophils, dendritic cells, macrophages, eosinophils, lymphocytes, and other cells, which are capable of producing a variety of cytokines, cytotoxic mediators including reactive oxygen species, serine and cysteine proteases, MMPs, membrane perforating agents, soluble mediators of killer cells, tumor necrosis factor alpha, interleukins, and interferons (55). Evidence indicates that these oncoproteins, especially E6 and E7 promote the expression of VEGF and other angiogenesis factors, such as IL-8, which can promote tumor angiogenesis through a HIF-1α/VEGF pathway in human cervical carcinoma cells and in non-small cell lung cancers (106, 107). A hypoxic state coupled with HPV infection favors an immunosuppressive environment in which activation of regulatory T cells and PD-L1 expressions occurs, enabling immune evasion of the tumor (108, 109). Together, these mechanisms make the HPV-HIF axis a potential target for targeted therapies.

HIF-1 can regulate some genes that are detrimental to the persistence of HPV-infected cells. One of them is interleukin-8 (IL8), which promotes inflammatory responses and angiogenesis (110).

From the onset of HPV infection through lesion development and ultimately progression to cancer, HPV can modulate the cellular response to hypoxia. One study indicates that HPV can increase HIF-1α levels through protein stabilization, resulting in increased expression of a subset of HIF-1 target genes, inferring that this increased expression may play a role in HPV disease progression (111). The HIF-1α protein contributes to progressive tumor growth, and it is feasible that this effect is synergistic with the HPV oncogene. The E6/E7 oncoproteins expressed in HPV cells mechanically reduce the induction of cellular senescence; therefore, increased expression of E6/E7 oncoproteins in HPV cells can reduce and suppress p53 transcriptional activity, such that the interaction between HIF-1α protein and HPV oncogenes can result in a series of gene expressions and lead to the development of invasive cervical cancer (112).

The PHD2-VHL-CUL2-ELOC axis represents the molecular mechanism by which cells respond to oxygen availability, post-translationally regulating the stability of hypoxia-inducible factor 1α (HIF-1α). PHD2 (domain 2 prolyl hydroxylase, also known as EGLN1) is an Fe²^+^- and 2-oxoglutarate-dependent dioxygenase that, under normoxic conditions, hydroxylates specific proline residues in the oxygen-dependent degradation domain of HIF-1α; this modification allows the factor to be recognized by VHL (von Hippel-Lindau protein), which, together with ELOC (elongin C) and ELOB (elongin B), assembles a substrate recognition complex that binds to CUL2 (culin 2) and RBX1 to form a functional E3 ubiquitin ligase, tagging HIF-1α for proteasomal degradation and thus preventing its accumulation as long as oxygen is available (113). Under hypoxia, the lack of oxygen as a co-substrate inactivates PHD2; HIF-1α escapes hydroxylation and degradation, becomes stabilized, translocates to the nucleus, and forms a heterodimer with HIF-1β to activate the transcription of genes involved in angiogenesis and metabolic reprogramming (114). In the context of cervical cancer, recent bioinformatics evidence suggests that the HPV-16 oncoproteins E6 and E7 may directly interfere with this pathway by interacting with VHL, CUL2, and ELOC within the E3 ligase complex, promoting the non-hydroxylation and non-ubiquitination of HIF-1α; which would lead to its aberrant stabilization even under normoxic conditions (pseudohypoxia), sustaining the Warburg-like metabolic reprogramming and tumor progression characteristic of cervical lesions associated with high-risk HPV. American Society of Hematology (115).

New evidence has shown that HPV16 infection promotes tumor progression through the activation of HIF-1α and associated metabolic reprogramming. Viral oncoproteins E6 and E7 regulate the PHD2-VHL-CUL2-ELOC-HIF-1α pathway, affecting the expression of key glycolysis genes such as GLUT1, LDHA and MCT4, promoting acidification of the tumor microenvironment and cell proliferation (115). Furthermore, HPV16-positive cervical cancer cells have been observed to display elevated levels of HIF-1α and glucose metabolism-associated genes compared to HPV-negative cells (105). Overexpression of vesicle-associated membrane protein 8 (VAMP8) in these cells increases autophagy through HIF-1α, further promoting tumor progression (116). The E6 oncoprotein contributes to the Warburg effect by stabilizing HIF-1α through inhibiting its interaction with VHL, thus enhancing metabolic reprogramming (117, 118). Finally, E6 also regulates PD-L1 expression through the miR-143/HIF-1α axis, allowing tumor cells to evade the immune response (119).

**Figure 2:**
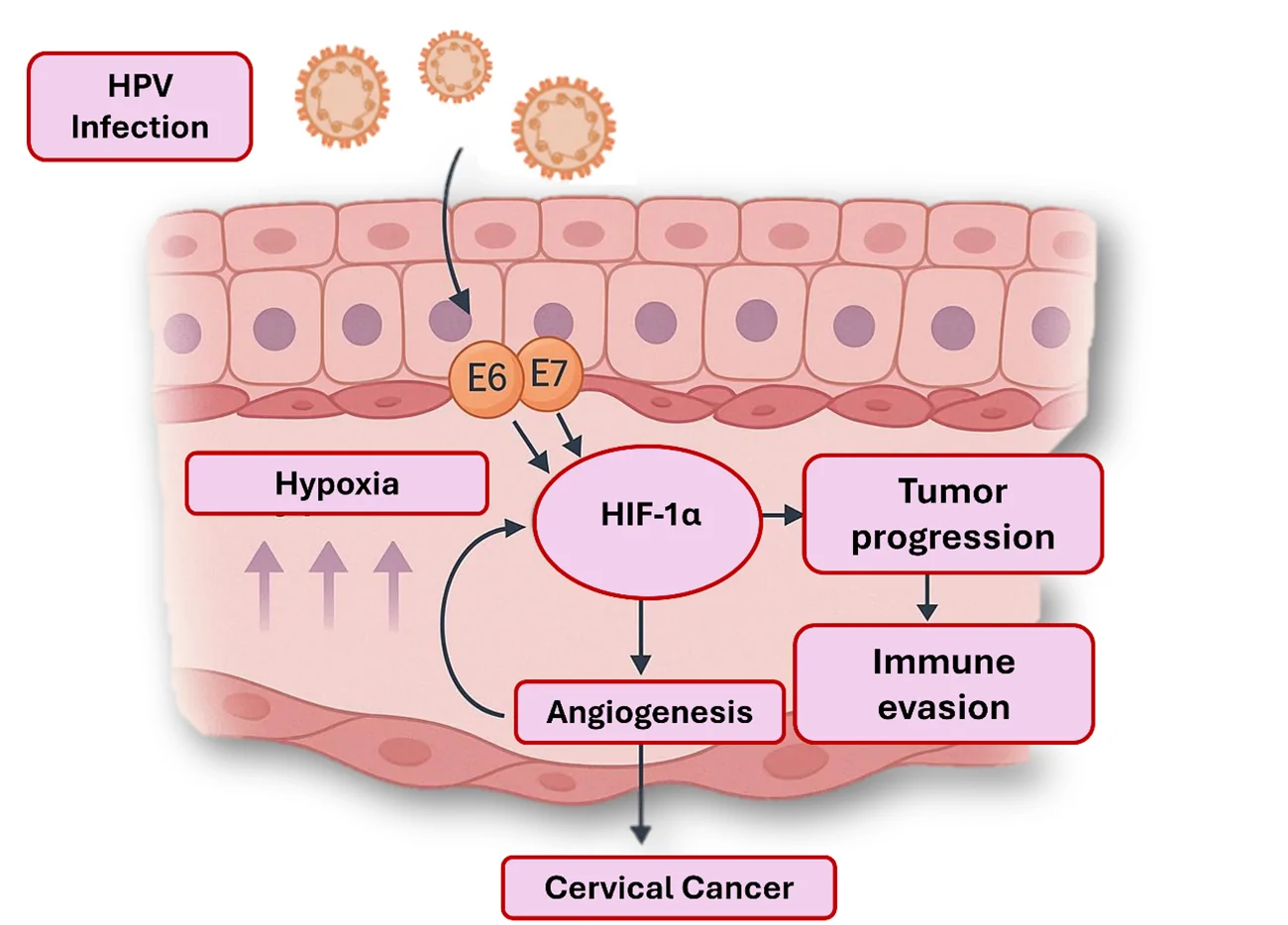
In the process of carcinogenesis, especially in cervical cancer where HPV plays a key role through the action of oncoproteins E6 and E7, tumor immune escape poses a significant obstacle to effective treatments. Hypoxic conditions, driven by inadequate blood supply as cancer progresses, activate key players such as HIF-1α orchestrating immune escape by upregulating molecules such as VEGF, promoting angiogenesis.

**Figure 3:**
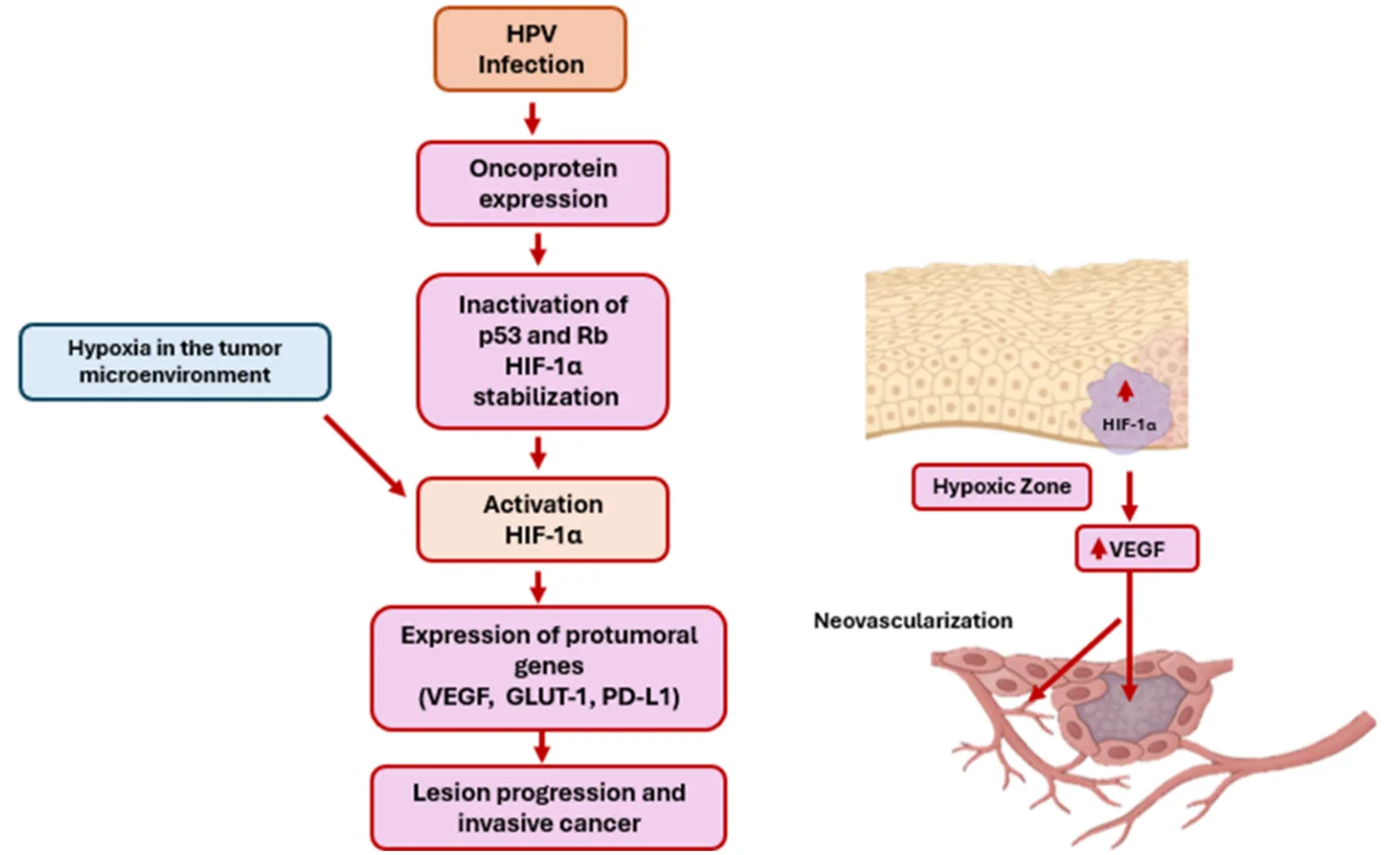
In active HPV infection, the E6 and E7 oncoproteins are expressed, triggering the PI3K/AKT/mTOR pathway through p53 degradation and inducing the retinoblastoma tumor suppressor gene. Under hypoxic conditions, the E7 oncoprotein increases the expression of hypoxia-inducible factor-1 alpha (HIF-1 alpha) through ROS, ERK1/2 and NF-κB, while E6 prevents HIF-1 alpha degradation, affecting cell proliferation, apoptosis, metabolism, immune responses, genomic instability, vascularization, neovascularization, invasion, and metastasis.

A study in transgenic mice determined that the combinatorial expression of the HPV16 oncogene and the gain of function of HIF-1α produced locally advanced cervical cancers that closely mimic the histopathological characteristics of locally advanced clinical disease (120). The effect of hypoxia on p53 was studied *in vitro* using assays on HPV16-positive cervical cancer cell cultures (SiHa, CaSki) at 21% O_2_ (normoxia) or 1% O_2_ (hypoxia) for 24 hours. It was concluded that, despite a negative regulation of HPV16 E6/E7 oncogene expression of up to 90% under hypoxia, p53 was not restored and even decreased to almost undetectable levels, while HIF-1α was easily induced, stimulating the transcription of VEGF (112) (**Figures 2**, **3**).

### Hypoxia-inducible factors and cervical intraepithelial neoplasia/cancer

In various types of cancer, such as CIN/Cancer, breast, head and neck, and sarcoma cancers regions of tumor hypoxia are described, producing an increase in the expression of HIF-1α and HIF-2α proteins in response (18). Comparative immunohistochemical studies showed that HIF-1α expression is low or absent in normal cervical epithelium, increasing in high-grade lesions (CIN2/3, carcinoma *in situ*) and even higher in invasive carcinoma. The positive expression rates of HIF-1α proteins, Yes-associated protein (YAP), and TAZ transcriptional coactivator (YAP/TAZ) in normal cervical tissues were lower than in CIN and cervical squamous cell carcinoma (CECC), suggesting an association with those signal pathways (115). When the clinical correlation of HIF-1α and YAP/TAZ expression was analyzed in normal tissues, CIN, and cervical squamous cell carcinoma (CSC), it was found that HIF-1α levels were significantly correlated in tumor tissue compared to normal epithelium and pre-invasive lesions. This suggests that HIF-1α significantly promotes the proliferation, invasion, and migration of cervical carcinoma cells by upregulating YAP/TAZ. Furthermore, it suggests that YAP/TAZ plays an important role in subclinical cervical carcinoma (SCCC) cells and that HIF-1α has therapeutic potential (88, 121) CIN/Cancer has certain characteristics that differentiate it from other solid tumors. Healthy lamellar epithelium, including cervical cells, has the characteristics of chronic hypoxic tissue and HIF-1 α levels increase significantly in advanced stages of CIN (122). In this regard, experimental and clinical studies in CIN/Cancer have associated HIF-1α overexpression with a series of aggressive biological phenomena. HIF-1α can increase cell migration and invasion, promoting epithelial-mesenchymal transition (EMT), favoring angiogenesis, and conferring resistance to apoptosis, which translates into easy local progression and metastatic spread. Regarding to this, clinical reports show that HIF-1α is involved in cancer progression, acting in early stages of CIN/Cancer and promoting survival under hypoxic conditions (123). These clinical studies have shown that HIF-1α is related to poor disease-free survival (DFS), overall survival (OS), tumor size, anemia (16) and prediction of metastasis-free survival (124) in cervical cancer. Studies from Birner et al. (125) indicate that HIF-1α expression is a powerful independent prognostic marker in early-stage of CIN/Cancer. Experimental studies conducted in cell lines and animal models of CIN/Cancer demonstrated that manipulation of HIF-1α alters tumor proliferation and metastatic capacity, and that HIF-1α can regulate oncogenic signaling networks such as YAP/TAZ that enhance malignant phenotypes (88, 121). In this way, the accumulation of HIF-1 α in the nucleus can regulate downstream genes through various mechanisms to promote tumor cell proliferation, invasion, energy metabolism, epithelial-mesenchymal transformation (EMT), and immune escape, resulting in tumor metastasis and poor patient prognosis. In this regard, nuclear HIF-1α expression was correlated with certain clinical variables such as tumor size, lymphovascular invasion, lymph node metastasis, and overall disease-free survival. It was found that HIF-1α-positive tumors showed greater lymphovascular invasion, lymph node metastasis, and larger tumor size, and patients with high HIF-1α expression had significantly lower overall survival (6, 126–128).

HIF-1α activates genes in the nucleus, inducing them to express factors involved in the carcinogenic effect of HIF-1α in CIN/Cancer (129). It has been reported that the expressions of HIF-1α and GLUT1 gradually increased from normal cervical tissue to CIN and subsequently to cervical cancer. HIF-1α was strongly associated with pathological differentiation, clinical stage, lesion size, and patient age, while GLUT1 was associated with lymph node metastasis (130). Overexpression of HIF-1 increased the endogenous expression of uPAR, a key receptor in the plasminogen activation system involved in extracellular matrix degradation and tumor migration/invasion. Conversely, the introduction of siRNA for HIF-1α decreased uPAR expression during hypoxia, indicating that hypoxia-induced upregulation of uPAR in CIN/Cancer is mediated by HIF-1. Analysis of cervical cancer tissues revealed uPAR expression, but not in normal cervix cells or CIN, suggesting that HIF-1-mediated upregulation of uPAR expression constitutes a mechanism of CIN/Cancer invasion during hypoxia (131). Recently, other proteins such as Survivin, which belongs to the inhibitor of apoptosis (IAP) protein family and is encoded by the BIRC5 gene, have been associated to HIF-1α. Survivin expression is considered a marker of CIN and high-risk human papillomavirus (HPV) infection, and a predictor of viral clearance and prognosis in cervical cancer (132). Another protein associated with HIF-1α expression is Erythropoietin (EPO). This protein is a glycoprotein produced in the kidney that stimulates erythropoiesis in response to hypoxia, under the control of HIF-1α. EPO and its receptor (EpoR) have been produced in various tumors, including cervical cancer. Previous study has demonstrated that the HIF-1α–EPO–EpoR system is activated in pre-invasive lesions and invasive cervical cancer. This suggests that increased HIF-1α, Epo and EpoR expressions play an important role in cervical carcinogenesis, tumor progression, and treatment resistance in hypoxic cervical cancers (133). Studies related to the expression of HIF-1α and its associated genes LIM domain containing 1 (LIMD1), von Hippel–Lindau (VHL), and VEGF in samples from healthy normal cervix, normal cervix adjacent to tumors, CIN, and cervical cancer, as well as two cell lines, reported that the coordinated dysregulation of LIMD1 and VHL by mutations or methylation generated abnormal stabilization of HIF-1α, even under normoxic conditions. This increase in HIF-1α could induce sustained activation of VEGF, stimulate tumor angiogenesis and promote the progression of intraepithelial lesions. Therefore, the LIMD1–VHL–HIF-1α–VEGF pathway is progressively dysregulated from normal cervical epithelium to invasive carcinoma, favoring angiogenesis and tumor survival (134). Studies analyzing the expressions of HIF-1α and MET in both cervical cancer cell lines and human normal epithelium, CIN 1-3, and invasive squamous cell carcinoma (ISCC), reported that HIF-1α and MET changed their expression pattern: in normal tissue, they were restricted to basal cells, but in dysplasia and carcinoma, they appeared in more superficial/differentiated cells, suggesting a role in tumor progression (90). MET encodes the c-Met tyrosine kinase receptor protein, but, when the MET gene undergoes mutations, such as amplification or sequence alterations, it can lead to uncontrolled cell growth that contributes to metastasis and treatment resistance in various cancers (135). Previous studies have identified VEGF (vascular endothelial growth factor) in pre-invasive cervical lesions as key elements and prognostic markers (55). Other studies have demonstrated the increased expressions of VEGF and HIF-1α.

An analysis of the available immunohistochemical evidence strongly supports a progressive though not always linear increase in HIF-1α expression as cervical tissue progresses from normal to intraepithelial neoplasia and, ultimately, to invasive carcinoma. In this context, a study that evaluated 30 samples of normal cervical tissue, 58 CIN samples, and 40 cervical squamous cell carcinoma samples using immunohistochemistry found that the HIF-1α positivity rate increased in a stepwise manner across these three categories, and that such positivity was also associated with a lower degree of tumor differentiation and with lymph node metastasis in the invasive carcinoma subgroup (121). For its part, a larger-scale study, which applied automated digital analysis of immunohistochemistry to 819 samples including 357 adjacent nontumor tissues, 209 CIN cases, 74 carcinomas *in situ*, and 179 invasive carcinomas, enabled a quantitative analysis of the expression profile of HIF-1α and other markers of hypoxia and metabolism (c-Met, carbonic anhydrase IX, GLUT1) across this same continuum (130).

In immunohistochemistry samples, both VEGF and HIF-1α had higher expression in CIN 2/3 and in invasive cancer, compared to the normal cervix. In this study, a weak correlation between the expression of VEGF and HIF-1α was obtained, implying that other regulatory mechanisms, in addition to HIF-1α, could be involved in the expression of VEGF during cervical carcinogenesis (136). Other studies showed that HIFs actively participate in angiogenesis by upregulating genes that influence the expression of VEGF and other proangiogenic factors, such as placental growth factor (PGF), platelet-derived growth factor β (PDGF-β), plasminogen activator inhibitor-1 (PAI-1), matrix metalloproteinases (MMP-2 and MMP-9), and angiopoietins (ANG-1 and ANG-2) all of which play a crucial role in carcinogenesis (137). It has been described that in several types of cancers, angiogenesis occurs only late in tumor progression, however, increased vascular density and production of angiogenic factors is an early event in the development of HPV-induced premalignant lesions and cervical cancer (138, 139).

Immunohistochemical scoring of HIF-1α in CIN evaluates tissue protein expression. The scoring relies on either the percentage of positive cells or an Allred/Immunoreactive (IRS) score (combining staining intensity and cell percentage), helping clinicians monitor the transition from precancerous lesions to invasive carcinoma. HIF-1α expression strongly correlates with cervical cancer progression; its levels rise to drive metabolic shifts and neoangiogenesis. Strong HIF-1α overexpression predicts poorer overall survival, shorter disease-free survival, and higher tumor-associated mortality in invasive cervical cancer (16, 121, 140, 141).

Another complementary finding analyzed the expression of VEGF and podoplanin in 234 samples of cervical squamous intraepithelial lesions stratified by histological grade: VEGF positivity was statistically significantly associated with the grade of the lesion, being more intense and diffuse in CIN2 and CIN3 compared with CIN1, whereas podoplanin expression showed the opposite pattern, with negative or focal staining being more common in CIN3 (M). Because VEGF is one of the best-characterized transcriptional target genes of HIF-1α, this angiogenic gradient observed as early as the preinvasive stage is consistent with early—though not exclusive—activation of the hypoxia response axis during the progression of intraepithelial neoplasia, before invasion into the stroma (142).

A study investigated the expression levels of miR-143, described as a tumor suppressor microRNA, in various cancer types to elucidate its role in the proliferation and apoptosis of cervical cancer cells and to determine whether its antitumor action is exerted through the downregulation of HIF-1α. Cases of normal cervical tissue, tissue with cervical intraepithelial neoplasia, and cancerous tissue were included. The results showed that overexpression of miR-143 significantly inhibited proliferation and promoted apoptosis in HeLa and SiHa cells. Furthermore, a reduction in miR-143 expression was found in cervical cancer cells compared to normal epithelial cells. Therefore, miR-143 could act as a tumor suppressor in cervical cancer, and its effect occurs through post-transcriptional repression of HIF-1α, reducing cell proliferation and increasing apoptosis, inferring that low expression of miR-143 and overexpression of HIF-1α could contribute to the progression of cervical cancer (143).

**Figure 4:**
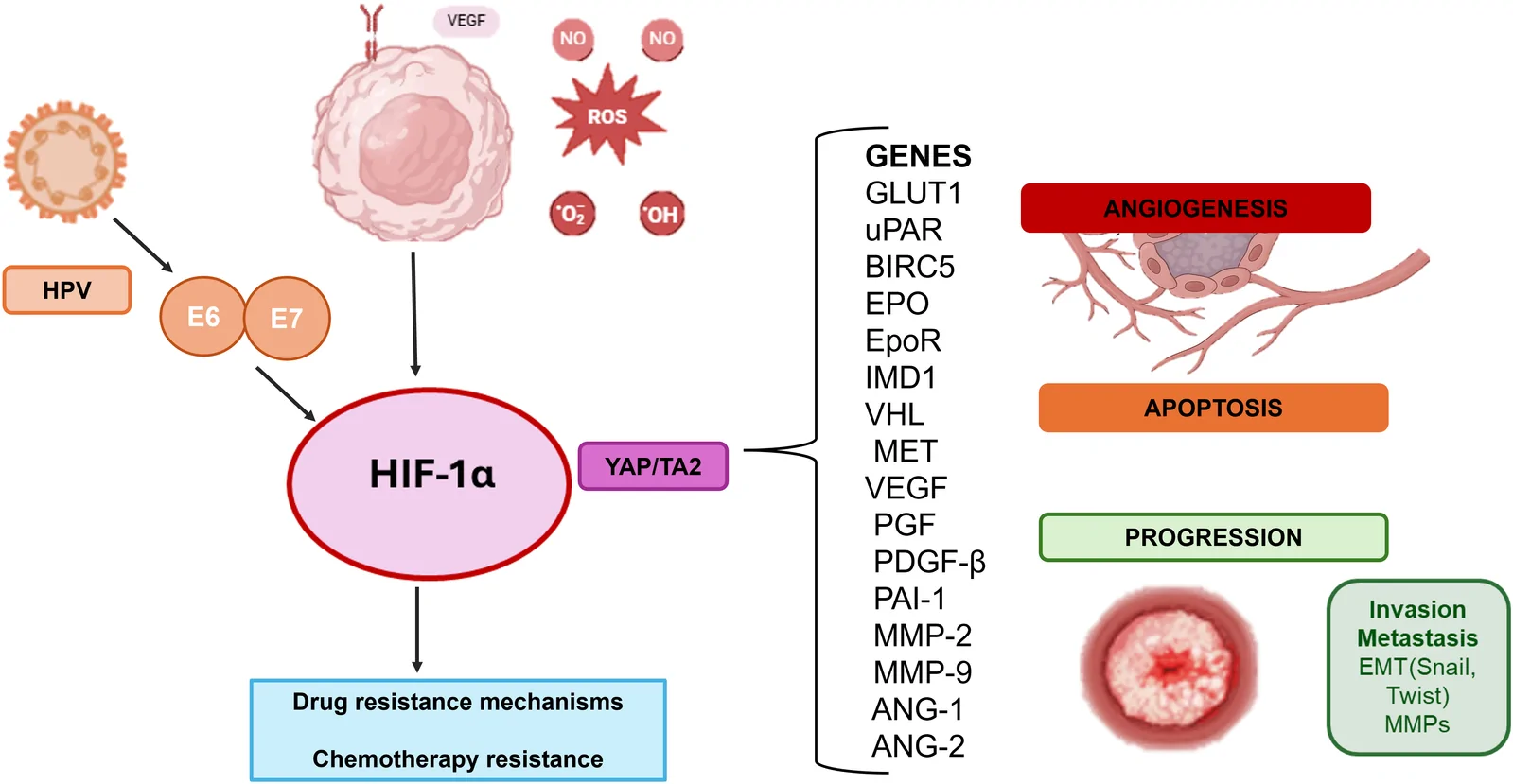
Genes used by hypoxia-inducible factor-1 alpha (HIF-1α) for the induction of progression in cervical intraepithelial neoplasia (CIN) and cervical cancer. HIF-1α is involved in the progression and invasion of CIN and cervical cancer mediated by different mechanisms that involve the expression of genes related to angiogenesis, apoptosis and progression such as GLUT1, uPAR, BIRC5, EPO, EpoR, LIMD1, VHL, MET and pro-angiogenic genes (VEGF, PGF, PDGF-β, PAI-1, MMP-2, MMP-9, ANG-1, ANG-2), and using pathways such as YAP/TA2. However, antitumor factors such as miR-143 and other antitumor factors can define the progression of this neoplasm.

**Figure 5:**
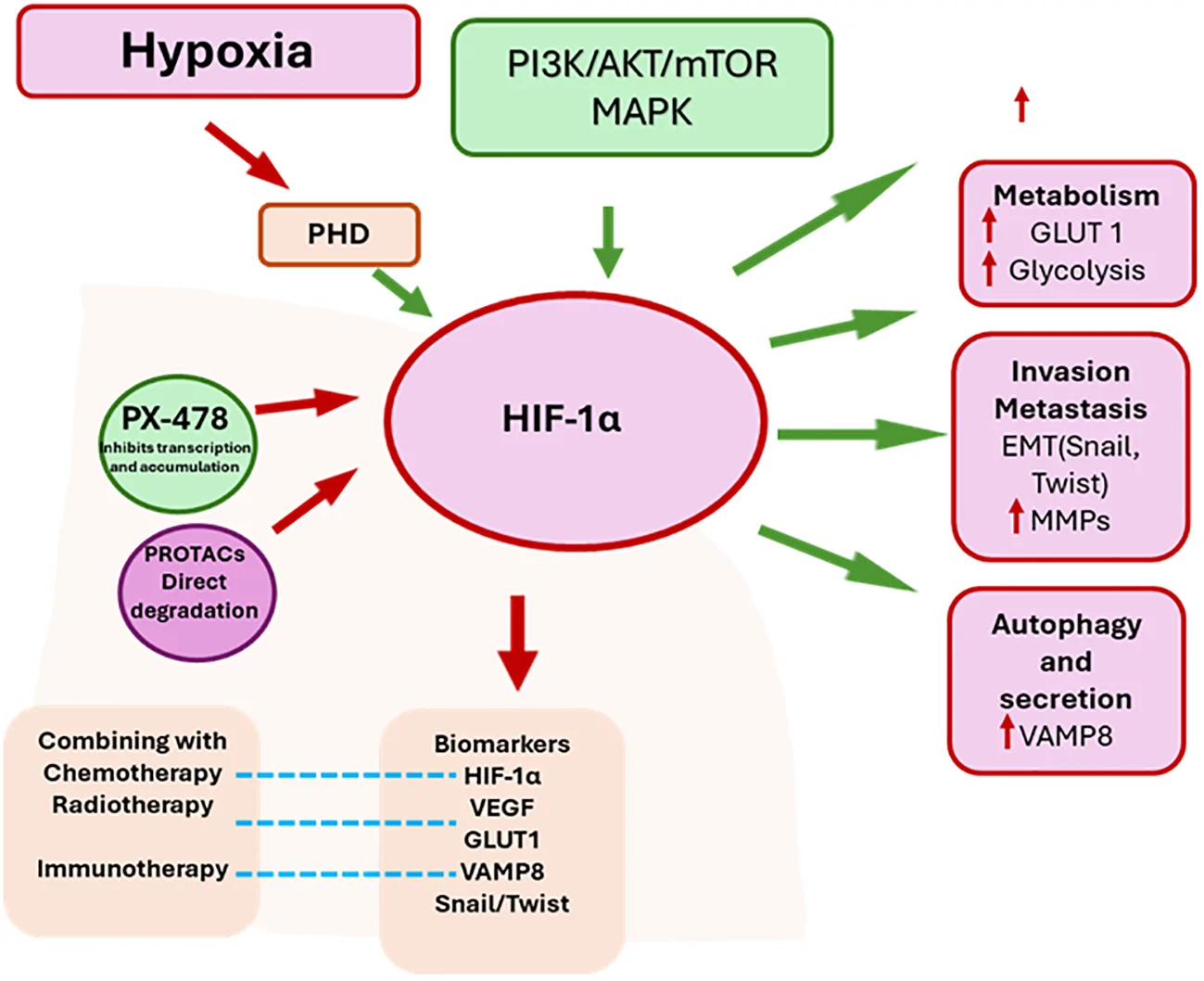
HIF-1α is activated under hypoxic conditions and through PI3K/AKT/mTOR and MAPK signaling pathways. Its activity promotes processes such as angiogenesis (↑VEGF), metabolism (↑GLUT1 and ↑glycolysis), invasion and metastasis (EMT with Snail and Twist, ↑MMPs), and autophagy and secretion (↑VAMP8). Inhibitors such as PX-478 reduce HIF-1α transcription and accumulation, while PROTACs induce its direct degradation. HIF-1α is also considered a useful biomarker in combination therapies with chemotherapy, radiotherapy, and immunotherapy, along with other associated markers such as VEGF, GLUT1, VAMP8, and Snail/Twist, key transcription factors that regulate the epithelial-to-mesenchymal transition (EMT). Green arrows indicate activation, red arrows indicate inhibition, and dotted arrows indicate therapeutic combinations.

As these data demonstrate, HIF-1α is involved in the progression and invasion of CIN and cervical cancer mediated by different mechanisms that involve the expression of genes related to angiogenesis, apoptosis and progression such as GLUT1, uPAR, BIRC5, EPO, EpoR, LIMD1, VHL, MET and pro-angiogenic genes (VEGF, PGF, PDGF-β, PAI-1, MMP-2, MMP-9, ANG-1, ANG-2), and using pathways such as YAP/TA2. However, the balance between antitumor factors (miR-143) and protumor factors can define the progression of this neoplasm (**Figures 4**, **5**).

Chronic hypoxia and intermittent hypoxia are differentiated by the duration and pattern of oxygen deprivation. Chronic hypoxia is continuous, prolonged exposure to low oxygen, leading to sustained physiological adaptations (e.g., higher red blood cell count). Intermittent hypoxia features rapid cycles of low and normal oxygen, which triggers oxidative stress, inflammation, and cellular dysfunction (43). Cervical intraepithelial neoplasia (CIN) progression is heavily influenced by its microenvironment. In this pathology while chronic hypoxia promotes cell dormancy and suppresses viral oncogenes, intermittent or cyclic hypoxia (cycling between oxygen deprivation and reoxygenation) drives malignant tumor phenotypes by maintaining viral E6/E7 oncogene expression, Sfueling inflammation, and causing treatment (20, 111, 144). Cellular hypoxia affects all crucial processes involved in tumor growth, invasion and metastasis. Metastasis is a multi-step complex process involving steps such as dissemination from primary tumor, entry into circulation, survival during circulation in matrix- deprived conditions and colonization of distant organs (145). During the metastaticy, cells may face varying extents of therapeutic attack, lack of nutrient and/or oxygen availability and other stressed conditions to which they dynamically adapt. During CIN/Cancer intermittent hypoxia increases HIF-1- α effect on angiogenesis by secreting important factors like VEGF (146–148). Tumor hypoxia can increase the cancer stem cells (CSCs) population through HIF-1α, specifically, under chronic hypoxia (2% O2, >24 h) (149). Intermittent hypoxia can also influence epithelial–mesenchymal transition (EMT), invasion and metastasis of cancer cells (150, 151). A small population of cancer cells in tumors tend to exhibit enhanced resistance against multiple therapies. It was observed that oxygen-deprived cells were three times more resistant to radiation therapy than well-oxygenated cells, when irradiated. As tumor microenvironment can have regions of chronic and cyclic hypoxia, they can produce persistent and/or transient radio-resistance, respectively (152). HIF-1α independent mechanisms may also affect the radioresistance of cells exposed to cyclic hypoxia. It has been reported radioresistance under cycling hypoxia with severe oxygen deficiencies (153). Inflammation is a hallmark of cancer and an important feature of tumor microenvironment (TME) that facilitates escape of cancer cells from the immune system (154). Regarding this, several factor have been reported increased during cyclic hypoxia such as amplified proangiogenic phenotype, higher THP-1 monocyte adhesion through NF-κB, increased PTGS-2, IL-6, CXCL1 and macrophage inflammatory protein (155), NF-κB mediated enhanced expression of prometastatic and proangiogenic factors (156), increased protumor effects of TAMs mediated by NRP-1, increase in NRP-1 levels and infiltration of CD206+ macrophages in tumor (157). These data summarize hypoxic conditions used to experimentally impose hypoxia *in vitro* and *in vivo* with respect to the three broad classes of hypoxia: acute, chronic and intermittent, and the different molecular mechanisms and phenomenon that are activated in these scenarios. HIF-1α and HIF-2α are known as master regulators of cellular hypoxic responses whose levels are dependent on various factors: the duration of hypoxia, O2 concentration and presence of hypoxia and reoxygenation cycles.

### Immune checkpoints

Immune checkpoints are pathways incorporated into the immune system. They are vital for modulating the quality of immune responses against pathogenic infection and maintaining self-tolerance in the peripheral tissues to minimise tissue damage (158). They are divided into two groups; co-stimulatory checkpoint molecules including CD27, CD28, CD137, ICOS, 4-1BB and OX-40 activate T cells, while co-inhibitory checkpoints like PD-1, CTLA-4 elicit inhibitory signals that prevent T cells activation to avoid excessive inflammation and autoimmune attack. However, in the tumour microenvironment, some of the inhibitory checkpoints are overexpressed and contribute to tumour-promoting immunosuppression, therefore the inhibition of negative immune checkpoint molecules represents a promising target for enhancing immune responses against tumour cells (159).

It is important to note the link between HIF signaling and immune evasion, which is mediated by immune checkpoints—a relationship that has been consistently established in multiple tumor models and PD-L1 (programmed death-ligand 1) has been identified as a direct transcriptional target of HIF-1α: its blockade under hypoxic conditions enhances T-cell activation mediated by myeloid-derived suppressor cells (MDSCs), demonstrating that intratumoral hypoxia, signaled through HIF-1α, constitutes an active mechanism of immunosuppression and not merely a metabolic epiphenomenon (160). In the context of cervical cancer, viral biology provides a second mechanism for amplifying this pathway: the integration of HPV DNA into the host genome, which is necessary for the process of malignant transformation. This process has been associated in the Cervical Cancer Genome Atlas with the amplification of the genes encoding PD-L1 and PD-L2, which formed the basis for incorporating PD-1 blockade into the treatment of this neoplasm (161).

The coexistence of two converging pathways leading to PD-L1 overexpression E6/E7-induced HIF-1α stabilization and gene amplification associated with viral integration offers a plausible mechanistic explanation, although not yet prospectively verified, for why cervical cancer responds to PD-1 blockade with relative independence from PD-L1 status as measured by conventional immunohistochemistry (162).

At the level of the tumor microenvironment, HIF-1α-mediated hypoxia also remodels the extracellular matrix via metalloproteinases, generates a disorganized VEGF-dependent vasculature, and promotes lactic acidification of the microenvironment three mechanisms that collectively hinder the infiltration of effector lymphocytes and undermine antitumor immune surveillance (163). This convergence of metabolic, angiogenic, and immunological pathways under the unified transcriptional control of HIF-1α provides the central rationale for considering this factor as a target for precision oncology that is potentially complementary to rather than an alternative to checkpoint inhibition.

### Therapeutic approaches

Hypoxia is an adverse feature of solid tumors, leading to metastasis and resistance to radiotherapy, chemotherapy, and possibly molecularly targeted drugs and immunotherapies (164). Physiologically, hypoxia is described as a stimulus for apoptosis (165, 166). However, the occurrence of hypoxic cervical cancers with a low fraction of apoptotic cells has been shown to be highly aggressive compared to hypoxic tumors with high apoptosis rates and non-hypoxic tumors (167, 168). Understanding the underlying mechanisms is vital for the development of new therapeutic alternatives and prognostic markers. In this regard, HIF-related mechanisms have long been a goal for cancer therapy, but it has been considered non-pharmacological. The discovery of a pharmacological pocket in HIF2α has led to the identification of small molecule inhibitors that allosterically disrupt its heterodimerization with HIF1β and can be safely used in humans (169). A better understanding of the HIF pathway in carcinogenesis increases the possibilities for intervention at the clinical level, which is why HIF is a potential target for therapeutic agents. These range from small molecules with the ability to inhibit HIF activity to gene therapies designed to modulate the expression of HIF-responsive genes. In addition, they provide valuable information that will allow the development of new diagnostic markers for early detection and prognosis of cancer (170). From a clinical perspective, an increase in tumor expression of HIF-1α correlates with advanced stages, larger tumor size, and increased risk of lymph node invasion, and therefore a worse prognosis in patients with cervical cancer; with some resistance or poor response to radiotherapy and chemoradiotherapy (129).Tumor hypoxia decreases the efficacy of radiation and promotes the activation of HIF-dependent cellular repair and survival pathways, contributing to therapeutic resistance (126, 170). Activation of HIF-1 causes the transcription of multiple genes involved in chemoresistance, such as genes that increase DNA repair capacity, block apoptosis, and promote drug efflux and cellular metabolism (171).

HIF-1α has been implicated in drug resistance mechanisms, promoting tumor growth, invasion, and resistance to chemotherapy. Control of HIF-1 expression is crucial as it can contribute to resistance to bevacizumab, used as an antiangiogenic agent targeting VEGF. However, HIF can promote blood vessel development through alternative pathways such as platelet-derived growth factor (PDGF) and fibroblast growth factor (FGF). As a result, tumors become more resistant and continue to grow and metastasize despite VEGF inhibition by bevacizumab (6, 63). Similarly, other factors such as transforming growth factor beta 2 (TGF-β2) have been linked to the activation of genes such as GLI2, which can increase intrinsic tumor resistance to chemotherapeutic drugs, and the overexpression of member 1 of the ATP-binding cassette subfamily B (ABCB1). This efflux drug transporter protein, ABCB1, is found in the cell membrane and excretes drugs from tumor cells, thereby reducing drug accumulation and inhibiting apoptosis that can be caused by chemotherapy, increasing resistance (6).

HIF-1α is frequently expressed in advanced stage cervical carcinomas and is considered a marker of poor response to therapy (124). In cervical cancer, although there is solid biological plausibility, robust clinical trials demonstrating the direct clinical benefit of HIF inhibition are still lacking; however, some preclinical studies and prognostic biomarkers support the clinical evaluation of these approaches (170, 172). Tests have been carried out for the pharmacological inhibition of HIF-1. In this respect, researchers have encountered certain limitations such as toxicity for new treatments targeting HIF-1 modulation. The findings demonstrate the efficacy of agents such as EZN-2968, Minnelide, and Acriflavine in modulating HIF-1α protein synthesis and destabilization, providing preliminary evidence of HIF-1α mRNA modulation and antitumor activity. However, further clinical studies are required (84). Expression of HIF-1α, VEGF-A, and Ki67 are important biomarkers in cervical neoplasia progression. Treatment with platinum-based neoadjuvant chemotherapy (PNACT) decreases expression of those molecules, suggesting that HIF-1α, VEGF-A, and Ki67 may be implicated in evaluating the efficacy of PNACT in locally advanced cervical cancer (126). Recent advances in the management of cervical cancer have focused on HIF-1α as a therapeutic target and on the identification of prognostic markers related to hypoxia. Thus, HIF-1α inhibitors such as PX-478 have been shown to reduce HIF-1α expression in HeLa cells and T cells, although they can also induce terminal exhaustion in mesoCAR (Chimeric Antigen Receptor) T cells. By decreasing HIF-1α levels, the activation of genes related to angiogenesis (VEGF), anaerobic metabolism (GLUT1, LDHA) and cell survival, which are promoted by hypoxia in tumors, is inhibited. In cervical cancer, PX-478 can reduce tumor cell proliferation and limit their adaptation to the hypoxic microenvironment (173). A retrospective multicenter study in patients with metastatic squamous cervical carcinoma and high HIF-1α expression treated with chemotherapy (platinum-paclitaxel) + bevacizumab, concluded that in metastatic cancer with high HIF-1α expression is associated with better patient survival if bevacizumab is added, while low HIF-1α expression correlates with a worse prognosis in patients treated with bevacizumab in combination with standard chemotherapy as initial therapy (174). Other drugs and technologies such as PROTAC (Proteolysis Targeting Chimera) which promotes selective protein degradation, demonstrating its potential to reduce HIF-1α expression and limit tumor progression (128) and combined strategies of HIF-1α inhibitors with immunological therapies (Programmed Death-1 (PD-1) blockers) to improve the antitumor response in the hypoxic microenvironment characteristic of cervical cancer (170) have been used in the treatment of cervical cancer.

There are potential molecular targets for monitoring and new therapies such as HIF-1α/ITGB1 (integrin beta 1). Bioinformatics analysis (GEO and TCGA) and *in vivo* validation determined that HIF-1α was a risk factor in univariate and multivariate analyses; and was correlated with immune infiltration and activation of TNF-α/NF-κB, PI3K/AKT, TGF-β, and JAK-STAT pathways. They also found that HIF-1α is associated with sensitivity to chemotherapeutic drugs, and *in vivo* models have confirmed that HIF-1α plays a crucial role in promoting the migration and invasion of cervical cancer cells, which could influence ITGB1 (128). Four key genes have been identified (ITGB1, CCL2, STRN3 and EDN1) potentially related to HIF-1α in the pathogenesis of cervical cancer, suggesting that these genes could influence cervical cancer progression through a set of biological functions and pathways, offering new insights into the etiology and therapeutic strategies for cervical cancer.

Certain fine regulatory pathways such as long non-coding RNAs (lncRNAs), microRNAs, and post-translational modifications that control HIF-1α in cervical cancer are emerging as potential biomarkers and complementary therapeutic targets. Several recent studies have identified pro-oncogenic lncRNAs that act through HIF-1α and as potential targets for modulating hypoxia-dependent signaling (175).

The addition of HIF inhibitors to the treatment regimen has demonstrated the ability to counteract resistance, with a synergistic effect in combination with chemotherapy, radiotherapy, targeted therapy, immunotherapy, and metabolic therapy in preclinical studies (170).

### Clinical translation

Metabolic fingerprints using16S rRNA gene sequencing and immunoassays, of. Vaginal microbiota and genital inflammation have been used to analyze patients with CIN and cervical cancer, and to reveal host and microbe contributions to the metabolome and to comprehensively assess the link between HPV, microbiota, inflammation and cervical disease (176).

Papilocare, a Coriolus versicolor-based vaginal gel, in repairing human papillomavirus (HPV)-related low-grade cervical lesions. Treatment with Papilocare has demonstrated a better clinical benefit than the conventional watchful approach in clinical practice for total and high-risk HPV patients in terms of its efficacy to treat HPV-related cervical lesions and to clear all HPV strains after a single 6-month period. It has demonstrated adequate safety and tolerability and confers additional benefits such as higher re-epithelization, stress reduction, and high treatment adherence (177).

HPV16/18 vaccination reduced the risk of HPV16/18-related CIN2+ lesions, but a substantial burden remained from non-vaccine high-risk types. The substantial protection against HPV16/18-related CIN2+ and the consequential shift in HPV genotype distribution of CIN2+ among vaccinated women underline the need for adaptation of screening strategies (178).

MiRNA may have roles in the pathogenesis of cervical cancer based on the increases or decreases in several specific miRNAs found in patients with this disease. The miRNA can affect HPV DNA replication shed more light on our understanding of the HPV life cycle and the mechanistic underpinnings of HPV induced oncogenesis. Also, miRNA processing proteins may be involved during early cervical cancer development. The E6 and E7 oncoproteins of HPV could induce the overexpression of DNA methyltransferase enzymes, which can catalyze the aberrant methylation of protein-coding and miRNA genes. Methods for diagnosis of cervical cancer include analysis of changes in the levels of specific miRNAs in serum and determination of aberrant hypermethylation of miRNAs. miRNAs are related on drug resistance and may be useful in combination therapy for cervical cancer with other drugs (179).

Recent advances in our understanding of T cell immunobiology have been particularly instrumental in informing therapeutic strategies to overcome mechanisms of tumor immune escape, and immune checkpoint blockade has emerged as one of the most promising therapeutic options for patients in the history of cancer treatment. Designed to interfere with inhibitory pathways that naturally constrain T cell reactivity, immune checkpoint blockade releases inherent limits on the activation and maintenance of T cell effector function. In the context of cancer, where negative T cell regulatory pathways are often overactive, immune checkpoint blockades have proven to be an effective strategy for enhancing the effector activity and clinical impact of anti-tumor T cells. Checkpoint inhibitors targeting CTLA-4, PD-1, and PD-L1 have yielded unprecedented and durable responses in a significant percentage of cancer patients in recent years (170).

Human papillomavirus (HPV)-associated intraepithelial neoplasia or cancers are ideal candidates for cancer immunotherapy since HPV oncoproteins, such as E6 and E7 proteins of high-risk HPVs, could be utilized as foreign antigens. It has been emphasized that therapeutic vaccines for HPV that are currently under development could be an important new tool in fighting against cervical cancer (180). In addition, in HPV-associated cancers as well as nonviral cancers, the cancer cells may evade host immunity through the expression of immune checkpoint molecules, downregulation of human leukocyte antigen, and activation of immune regulatory cells. Some promising studies using animal models and early human clinical trials raised a possibility that such combinations may be efficacious in regressing HPV-associated cancers (181). Blocking the expression of the FEN1 (Flap Structure-Specific Endonuclease 1) gene is a suitable target for preventing the progression of CIN to cervical cancer (182). Specific inhibitors of SIRT1 (Ex527) suggest probable application of sirtuin inhibitors as therapeutic targets in CIN (183). Targeting Tim-3 and PD-1 pathways reverses T cell exhaustion and restore anti-tumor immunity (184). Immunotherapies targeting the TIM3/PD-1/PD-L1 pathway have shown success in cervical and vulvar squamous cell carcinomas (185). The expression of the T-cell activation suppressor-immunoglobulin variable region (VISTA) in the development of cervical squamous cell carcinoma (CSCC) has been reported and can be used as an independent predictor of CSCC prognosis and can provide a strong basis for the treatment of CSCC with immune checkpoint inhibitors (186). These therapeutic insights, based on the pathogenesis of CIN and cervical cancer, provide a range of therapeutic options for treating CIN and cervical cancer.

Unlike traditional methods that group cancers based on where they are in the body, precision oncology looks at the specific genetic changes or proteins that cause the cancer to grow. These changes often can be treated with drugs designed to target them. It has been identified the molecular fingerprints of various cancers which allow cancers to be divided into various types and subtypes. It has been discovered that different cancers in different parts of the body can have a lot in common at molecular level. From this new perspective emerges an exciting era in cancer research called precision oncology, in which doctors are choosing treatments based on the DNA signature of an individual patient’s tumor. Using advanced technology to analyze large number of women with CIN and cervical cancer could refine diagnosis, identify inherited cancer susceptibility, or guide treatment for these patients (187, 188).

The role of HIF-1α has been studied for many years; **Table 1** summarizes key findings that have emerged since the 2000s. HIF-1α acts as the central molecular hub linking tumor hypoxia to disease progression and treatment resistance in cervical cancer. Its expression progressively increases from normal cervical tissue through intraepithelial neoplasia to invasive carcinoma, activating gene programs involved in glycolysis, angiogenesis, and invasion that correlate with advanced FIGO stage, lymph node metastasis, and poorer overall survival (**Table 2**).

**Table 1:** Clinical, molecular, and therapeutic implications of HIF-1α expression in the tumor microenvironment.

Dimension	Finding
Biological role	HIF-1α constitutes the central molecular axis linking tumor hypoxia to progression and treatment resistance in cervical cancer by activating gene programs involved in glycolysis, angiogenesis, and invasion (via the YAP/TAZ and ITGB1/CCL2/STRN3/EDN1 pathways).
Biological expression	HIF-1α progressively increases in normal cervical tissue, cervical intraepithelial neoplasia (CIN), invasive carcinoma, and correlates with advanced FIGO stage, lymph node metastasis, and lower overall survival.
Systemic treatment response	The expression of this marker predicts a poorer response to platinum-based neoadjuvant chemotherapy; its levels decrease significantly in patients who respond to treatment. Therefore, it could serve as a negative predictive marker and a monitoring marker.
Resistance mechanisms	Under prolonged hypoxia, HIF-1α and HIF-2α not only induce angiogenesis but also upregulate cytoprotective autophagy and HOTAIR-mediated radiation resistance. Notable exception: in highly glycolytic tumors, the mTOR/HIF-1α/c-Myc/PKM2 pathway was paradoxically associated with increased sensitivity to cisplatin.
Impact on the response to radiation therapy	Elevated expression prior to treatment consistently predicts a poorer response to radical radiation therapy, serving as an independent prognostic biomarker in advanced stages (IIB–IVA), and predicting a poorer response to radical radiation therapy due to hypoxia-mediated radioresistance.

**Table 2:** HIF-1α in HPV infection, precursor lesions, and invasive cervical cancer: evidence and treatment resistance.

*HIF-1α in HPV infection, precursor lesions, and invasive cervical cancer*			
Year	Clinical context/stage	Scientific finding	Reference
2009	High- and low-risk HPV infection (preclinical stage)	Complete HPV31 genomes (and, to a lesser extent, low-risk HPV11) stabilize HIF-1α under chemical hypoxia (DFO). Both E6 and E7 independently enhance this induction, indicating an early mechanism not restricted to oncogenic genotypes.	(111)
2014	HPV16 infection (E6/E7) metabolic reprogramming	HPV16 E6 oncoprotein complexes with HIF-1α and attenuates the VHL–HIF-1α interaction, blocking ubiquitination and proteasomal degradation, thereby sustaining the Warburg effect in infected cells.	(103)
2024	HPV16 E6/E7 PHD2-VHL-CUL2-ELOC axis	Bioinformatic analysis shows that E6 and E7 interfere with the E3 ubiquitin ligase complex (VHL-CUL2-ELOC), increasing HIF-1α stability and downstream target gene expression (GLUT1, LDHA, CAIX, MCT4, BSG) in cervical cancer.	(115)
2022	Cervical intraepithelial neoplasia (CIN, precursor lesion)	Positive HIF-1α, YAP, and TAZ expression rises progressively from normal cervical tissue through CIN to cervical squamous cell carcinoma (CSCC), correlating with lower differentiation and lymph node metastasis.	(121)
2000	Early-stage invasive cervical cancer (pT1b)	Immunohistochemical HIF-1α overexpression in 91 patients was independently associated with shorter overall and disease-free survival, establishing it as a strong prognostic marker.	(125)
2003	Cervical cancer treated with curative radical radiotherapy	Strong/moderate HIF-1α expression was associated with only partial response to radiotherapy and shorter progression-free survival, independent of the HPV genotype present.	(13)
2003	Cervical cancer treated with radical radiotherapy	HIF-1α was detected in 94% biopsies and was an independent factor for overall survival on multivariate analysis, with a weak inverse correlation to pretreatment hemoglobin.	(189)
2004	Stage IIIB cervical squamous carcinoma, radiotherapy alone	HIF-1α expression specifically predicted metastasis-free survival after radiotherapy alone, pointing to its role as a biomarker of distant spread rather than locoregional control.	(124)
2020	Locally advanced cervical cancer, neoadjuvant chemotherapy (NACT)	In stage IIB–IIIB patients, elevated pretreatment HIF-1α expression was associated with poorer response to platinum-based NACT and worse overall survival, functioning as an independent predictive marker.	(190)
2025	HPV infection (E6/E7)	The E6 and E7 oncoproteins of high-risk HPV act as foreign antigens, making virus-associated neoplasms ideal candidates for immunotherapy	(180)
2019	Cancer	Cancer cells evade the immune system by expressing checkpoint molecules, downregulating HLA antigens, and activating regulatory cells; combination therapies in animal models show potential for reversing these tumors.	(181)
2025	Cervical intraepithelial neoplasia	Inhibiting the expression of the FEN1 (endonuclease 1) gene has been identified as a therapeutic target that prevents cervical intraepithelial neoplasia (CIN) from progressing to cancer.	(182)
2025	Cervical intraepithelial neoplasia and cancer	The use of advanced technology to analyze the molecular mass profile of women with CIN and cervical cancer makes it possible to refine diagnosis, identify hereditary susceptibility, and guide personalized treatments.	(187, 188)
HIF-1α and cervical cancer treatment			
Year	Therapeutic aspect	Scientific finding	Reference
2020	Predictor of neoadjuvant chemotherapy response	Low pretreatment HIF-1α levels were associated with greater sensitivity to platinum-based neoadjuvant chemotherapy in locally advanced cervical cancer (stages IIB-IIIB); elevated expression predicted worse overall survival	(190)
2024	Interference with cellular immunotherapy	The HIF-1α inhibitor PX-478 reduced the proliferation and cytotoxic function of anti-mesothelin CAR T cells in cervical cancer models, showing that HIF-1α signaling also conditions the efficacy of adoptive immunotherapy.	(173)
2024	Chemical optimization of HIF-1α degraders	The development of next-generation HIF-1α PROTACs aims to overcome the specificity limitations and resistance susceptibility of the small-molecule inhibitors used so far in oncology.	(128)
2025	HIF1A-AS2/Cervical cancer	The long non-coding RNA (lncRNA) HIF1A-AS2 acts as an oncogene by regulating malignant phenotypes in cervical cancer; therefore, HIF1A-AS2 could be a viable and promising therapeutic target for the treatment of cervical cancer.	(175)
2024	Emerging therapeutic strategies that target HIF-1	It describes emerging therapeutic strategies targeting HIF-1, as well as the significance and regulation of HIF-1 pathways in cancer.	(172)
2023	Block the tumor defense mechanism triggered by a lack of oxygen	It proposes combating resistance to cancer treatments by inhibiting the hypoxia-inducible factor (HIF) in conjunction with current therapies. This combination strategy aims to block the tumor defense mechanism activated by a lack of oxygen, thereby improving the efficacy of chemotherapy, radiation therapy, and immunotherapy.	(170)
HIF-1α and therapeutic resistance			
Year	Mechanism	Scientific finding	Reference
2020	Aumiddlehagy and cisplatin resistance	HIF2A overexpression reduced cisplatin sensitivity in cervical cancer by inducing excessive aumiddlehagy; pharmacological aumiddlehagy inhibition (3-methyladenine) partially reversed this effect *in vivo*.	(191)
2003	Correlation Between HIF-1α Expression and Treatment Outcome in Cervical Cancer Treated with Radical Radiation Therapy.	Strong association between hypoxia-inducible factor-1 alpha (HIF-1α) and the efficacy of radical radiation therapy for cervical cancer. Higher levels of this protein are associated with poorer treatment outcomes.	(189)
2024	HIF-1α and Survival	High HIF-1α expression is associated with better survival in patients with metastatic squamous cell carcinoma of the cervix receiving first-line treatment with chemotherapy and bevacizumab.	(174)
2023	Metabolic reprogramming and chemoresistance	HIF-1α signaling shifts tumor metabolism toward glycolysis, generating an antioxidant environment (NADPH, glutathione) that protects cervical cells from chemotherapy- and radiation-induced cytotoxicity.	(126)

*In vitro* studies by Nakamura et al. (111) showed that the HPV genome even that of low-risk genotypes is sufficient to stabilize HIF-1α in response to a hypoxic stimulus, placing this phenomenon prior to any overt transformation. Guo et al. (103) and, more recently, Arizmendi-Izazaga et al. (115) clarify that the mechanism is as follows: the E6 and E7 oncoproteins do not directly induce HIF-1α, but rather interfere with its degradation pathway in t1α but-VHL-CUL2-ELOC axis, such that a factor that under normal conditions would have a half-life of minutes accumulates and reprograms cellular metabolism toward the aerobic glycolysis characteristic of the Warburg effect.

This same pattern of progressive accumulation is observed across the entire histological spectrum. The study by Abudoukerimu et al. (121) compares normal tissue, NIC, and squamous cell carcinoma within the same series and finds an increasing gradient of HIF-1α that accompanies and is likely promotes, via YAP/TAZ the loss of differentiation. This is clinically relevant because it suggests that HIF-1α is not only present at a late stage in tumor hypoxia but also acts as a key player, participating from the precursor lesion onward in the selection of more aggressive clones.

In invasive cancer, Birner et al. (125) found that immunohistochemical overexpression of HIF-1α, even in early stages (pT1b), independently predicts poorer overall and disease-free survival a finding that has stood the test of time reasonably well.

Perhaps the most useful aspect for clinical practice is that concerning treatment resistance. Both Bachtiary et al. (13) and Burri et al. (189), in independent studies of cohorts treated with radical radiation therapy, agree that strong expression of HIF-1α is associated with only a partial response and with poorer progression-free survival, while Ishikawa et al. (124) add an important nuance by showing that, in stage IIIB, the marker primarily predicts the development of distant metastases rather than locoregional tumor control. Another study (190) suggests that tumor hypoxia limits the efficacy of conventional and neoadjuvant platinum-based chemotherapy through mechanisms that are at least partially shared downregulation of apoptosis, selection of resistant subclones, and reduced generation of reactive oxygen species dependent on molecular oxygen.

The study by Jiang et al. (191) demonstrates that overexpression of HIF-2α (HIF2A) reduces sensitivity to cisplatin in cervical cancer by inducing excessive autophagy, confirming that HIF subunits act as potent mechanisms of tumor resistance.

The precision oncology approach has revealed that cancers in different anatomical locations may share common biological signatures and, therefore, respond to similar targeted therapies based on each patient’s DNA profile. In the context of gynecologic pathology, the implementation of advanced technologies for large-scale molecular analysis in women with cervical intraepithelial neoplasia (CIN) and cervical cancer ushers in a promising era; this mapping not only refines diagnostic accuracy and detects hereditary susceptibility but also optimizes the design of personalized and targeted therapeutic strategies (187, 188).

### Current limitations

There are currently limitations regarding the significance and reliability of studying HIF-1α in patients with CIN or cancer. Laboratory testing for HIF-1α lacks standardization; furthermore, CIN progression may occur via pathways independent of HIF-1α, and the presence of this molecule does not provide predictive values ​​for disease progression. Variability exists across laboratory protocols regarding tissue fixation and immunohistochemical standardization, which influences the quantification of HIF-1α in tissues. Differences in result interpretation must also be considered, as well as the fact that HIF-1α expression is often transient, driven by changes in the neoplastic microenvironment, rather than representing a stable marker of the premalignant state. It should be noted that HIF-1α can be induced in tissues by stimuli other than hypoxia; for instance, viral oncoproteins (such as HPV E6/E7) and growth factors can stabilize HIF-1α within the tissue. Although HIF-1α expression increases during the transition from normal tissue to CIN and invasive cancer, baseline levels do not reliably predict which specific CIN lesions will regress or progress.

## Conclusion

Hypoxia-inducible factor 1 alpha (HIF 1α) represents a critical factor of great importance in the pathophysiology of cervical cancer, acting as an inducer of the tumor response in hypoxic microenvironments. In neoplastic tissues of the cervix, the hypoxic state not only induces HIF 1α stabilization, but also triggers a transcriptional cascade that activates a series of mechanisms especially tumor angiogenesis, through the regulation of key genes such as VEGF that enable tumor growth and infiltration, promotes an aggressive phenotype, increasing proliferation, invasiveness and metastasis by inducing the production of mediators such as IL 8 (CXCL8), which favors cell migration and MMPs activity. Through induction of anaerobic glycolysis, GLUT 1 and glycolytic enzymes are overexpressed to adapt the cell to oxygen deficit and maintain energy production. HIF-1α can integrate hypoxemic signals with metabolic, angiogenic and pro-invasive modulations, functioning as a possible global controller of the aggressive tumor phenotype, therefore its overexpression is not only related to a hypoxic state but also to oncogenic activation in advanced stages of cervical cancer. HIF-1α is currently considered a prognostic marker and constitutes an emerging therapeutic target with possibilities to improve the response to conventional treatments and halt metastatic progression. However, it is necessary to continue its study and increase the evidence of clinical applicability and follow-up over time in patients diagnosed with cervical pathology.

A synthesis of the available evidence suggests that HIF-1α stabilization in cervical cancer should not be understood solely as a late adaptive response to hypoxia generated by macroscopic tumor growth, but rather as an event that can be triggered in parallel with the transforming HPV infection, mediated by the E6 and E7 oncoproteins through interference with the PHD2-VHL-CUL2-ELOC axis. The expression gradient observed across the normal-CIN-carcinoma continuum, for both HIF-1α and its target gene VEGF, is consistent with this hypothesis, although most available studies are cross-sectional rather than longitudinal follow-ups of individual lesions, which prevents us from establishing with certainty whether HIF-1α expression in a given CIN2 or CIN3 independently predicts its progression to invasive carcinoma versus its persistence or regression.

This limitation highlights a significant research gap, necessitating prospective cohort studies that evaluate HIF-1α expression in biopsies covering the CIN2–3 spectrum with documented clinical follow-up, similar to the designs already used for other biomarkers of progression in intraepithelial neoplasia, in order to determine whether HIF-1α can be incorporated as an auxiliary marker for risk stratification alongside HPV genotyping and established histological markers such as p16.
